# Can alternative liver function scores facilitate the establishment of an indication for radioablative therapy in patients with hepatocellular carcinoma?

**DOI:** 10.1007/s00432-022-04411-5

**Published:** 2022-10-16

**Authors:** Dominik Ott, Ahmed Gawish, Anke Lux, Constanze Heinze, Thomas B. Brunner, Peter Hass

**Affiliations:** 1 Department of Radiation Oncology, University Hospital Magdeburg, Leipziger Strasse 44, 39120 Magdeburg, Germany; 2 Institute of Biometry and Medical Informatics, University Hospital Magdeburg, Magdeburg, Germany; 3 Department of Radiology and Nuclear Medicine, University Hospital Magdeburg, Magdeburg, Germany; 4 Department of Radiation Oncology, University Hospital Graz, Graz, Austria; 5 Department of Radiation Oncology, Helios-Klinikum Erfurt, Erfurt, Germany

**Keywords:** ALBI, IBI, Brahytherapy, HCC, Radioablative therapy, Liver malignancy

## Abstract

**Background and purpose:**

ALBI and IBI are new scores to evaluate the liver function in patients with hepatocellular carcinoma (HCC). The purpose of this study was to evaluate the prognostic abilities of those scores in patients treated with interstitial brachytherapy (iBT).

**Materials and methods:**

190 patients treated with iBT between 01.01.2006 and 01.01.2018 were included in this study. The clinical target dose was 15 Gy. The patients were all in Child–Pugh stadium A or B and across the Barcelona Clinic Liver Cancer (BCLC) Stages 0–C. Retrospectively ALBI and IBI were calculated pre- and post-therapeutic until 6 months after iBT. Hazards ratios were calculated, and p values corrected using the false discovery rate according to Benjamini and Hochberg.

**Results:**

The median overall survival was 23.5 months (CI 19–28.5 months), and the median progression-free survival was 7.5 months (CI 6–9 months). Elevated ALBI showed a significantly higher risk to die with a hazard ratio (HR) of 2.010 (ALBI 2 vs. 1) and 4082 (ALBI 3 vs. 1), respectively. The IBI did also show a higher risk with an HR of 1.816 (IBI 1 vs. 0) and 4608 (IBI 2 vs. 0), respectively. Even 3 months after therapy elevated ALBI and IBI showed poor overall survival. Concerning progression-free survival, ALBI and IBI could not provide any relevant additional information.

**Conclusion:**

ALBI and IBI are useful tools to predict the overall survival in patients treated with iBT and might be helpful to assign the patients to the appropriate therapy.

## Introduction

Primary liver cancer is the 6th most common cancer in the world and has the 4th highest mortality. For men, it even has the 2nd highest cancer-associated mortality. Around 75–85% of all liver malignancies are hepatocellular carcinoma (HCC) (Bray et al. 2018). Even though most patients with HCC are from traditional high-risk regions like Asia, the incidence and mortality are rising in North America and parts of Europe (Kulik and El-Serag 2019). At the same time, new therapy options are getting more common, but there is still a problem to evaluate the prognosis for patients with HCC easily and reliably. The Child–Pugh Score (CP) is widely used to assess the liver function of patients with all kinds of liver diseases, but has never been validated for patients with HCC. Additionally, some criteria like ascites and encephalopathy are subjective. Furthermore, many patients are categorized as CP grade A which makes it impossible to differentiate between those patients (Johnson et al. 2015). The Barcelona Clinic Liver Cancer (BCLC) staging system also uses the CP score and other subjective data. The newly presented albumin–bilirubin grade (ALBI) uses albumin and bilirubin levels in the blood as objective data (Johnson et al. 2015). It has been verified and compared to the CP score in early HCC stages for patients receiving a radiofrequency ablation (RFA) (Oh et al. 2017) and resection (Ma et al. 2016; Wang et al. 2016; Li et al. 2017). It has also been shown to be reliable across all BCLC stages for patients receiving transarterial chemoembolization (TACE) (Pinato et al. 2017). The inflammation-based index (IBI) has been published further as an objective and easy way to predict the survival of patients with HCC, and has been shown to predict a better survival for patients undergoing TACE (Pinato et al. 2012; Pinato and Sharma 2012). Gkika et al. (2018) showed a better survival for patients with lower IBI undergoing stereotactic body radiotherapy, but not for lower ALBI. This retrospective study aimed to evaluate the prognostic value of the ALBI grade and the IBI score for overall survival (OS) and progression-free survival (PFS) for patients receiving interstitial brachytherapy (iBT) to show if those scoring systems can be used for clinical assessment of patients with HCC.

## Materials and methods

**Fig. 1:**
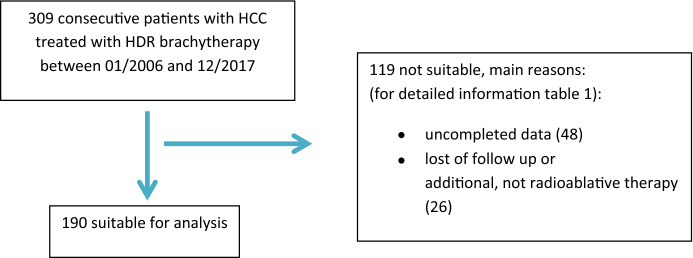
Flow diagram according to Moher (Moher et al. 2001)

All HCC patients first time treated with iBT between 01.01.2006 and 31.12.2017 were primarily considered. Of those 309 patients, 190 were suitable for this retrospective study (Fig. 1).

Most of the excluded patients (48) had missing data and missing follow-up observations or additive therapy at the same time (26). Patients were also excluded if the time between two brachytherapies was > 6 weeks, insufficient radiation due to suboptimal catheter placement or oligonodular tumor configuration causing a change in therapy or a repetition of the iBT, liver transplantation took place in the course, repetitive iBT was necessary, or other active malignancies were present at the same time. If patients had another therapy > 6 weeks before iBT they were included. The diagnosis of HCC was based on imaging and/or histopathological analysis according to Bruix and Sherman (2011). A contrast-enhanced CT or MRI was performed before therapy and at follow-up observations. Blood samples were also collected and analyzed. The follow-up observations were scheduled every 3 months after therapy. Due to the higher risk for tumor progression or complications, some patients had an untimely follow-up, and some observations did not take place, because patients canceled the appointments for different reasons. If a follow-up observation did not take place, it was not considered in the analysis (Table 1). The relevant laboratory parameters to calculate the scores were measured immediately before and after therapy and at the follow-up examinations after 6 weeks, 3 months, and 6 months. The ALBI and IBI score were calculated according to Johnson et al. (2015). Before therapy and at the follow-up examinations, the Child–Pugh score, Karnofsky performance status, and ECOG stage were determined. The BCLC stage was only evaluated before therapy. Data after a change of therapy were not recorded. Date of death of 15 patients was not listed in the clinical database, but was collected, if possible, with data from the State cancer registry. Pre-existing illnesses were extracted from earlier reports and pre-therapeutic examinations. Adverse events were recorded according to the Common Terminology Criteria for Adverse Events v5.0. The iBT was carried out with a 10 Ci iridium-192 source in an afterloading procedure. Catheter placement was CT or MRI-controlled. A detailed description can be found in Mohnike et al. (2016). The target dose was 15 Gy.

**Table 1:** Exclusion criteria

Exclusion criteria	Number of patients
No/irregular follow-up examinations or very bad documentation	48
Other locally ablative therapy at the same time like RFA or TACE	26
Two-timed iBT with an interval > six weeks	3
Insufficient radiation due to suboptimal catheter placement or oligonodular tumor configuration causing a change in therapy or a repetition of the iBT	3
Liver transplantation in the course	10
Complications during iBT causing death of the patient	2
Three-timed iBT	9
iBT not done	5
Other symptomatic or progressive malignancy	9
iBT in transplanted liver	1
First iBT before 2006	1
No clear HCC diagnosis	1
Missing date of death	1

### Statistical analysis

Statistical analysis was performed using SPSS 26 (IBM Corp., Armonk, NY, USA). The overall survival was measured from the first day of iBT until death. The progression-free survival was also calculated from the start of therapy until detection of progression in MRI/CT. For patients without progression before death, the date of death was used for calculation, and for patients who were still alive, the date of the last follow-up-observation with MRI/CT was used to calculate the PFS. A Cox regression was used to calculate the hazards ratio and the Kaplan–Meier procedure was used to illustrate the data. Patients who had no progression or who were still alive were censored according to the instructions of the procedures. The level of significance was 5% (*p* = 0.05). ALBI and IBI were used until 6 months after iBT. The increase or decrease of ALBI or IBI was calculated between pre- and post-therapeutic values. Three months after therapy, an increase was calculated in comparison to pre-therapeutic values, and a decrease compared to post-therapeutic values. We chose this procedure due to a low number of patients with high pre-therapeutic or low post-therapeutic ALBI or IBI levels. Because of the high number of tests for significance, the False Discovery Rate (FDR) according to Benjamini and Hochberg (1995) with an FDR of 5% was used to reduce the number of false-positive results. *p* Values were corrected with the formula *p*’ = p*(m/k) where *p*’ is the corrected *p* value, *p* is the original *p* value, m is the number of independent tests, and k is the position of the original *p* value sorted in ascending order (Jafari and Ansari-Pour 2019).

## Results

A total of 190 patients were included in our analysis. Overall, there were 375 lesions of which the treatment of 286 was described. 158 patients (83.2%) were male and 32 (16.8%) were female. 146 (76.8%) had cirrhosis of the liver. Eleven patients were infected with hepatitis B (7.5% of patients with cirrhosis, respectively, 5.8% of all patients), 22 (15.1% of patients with cirrhosis, respectively, 11.6% of all patients) had hepatitis C in which 3 patients had both. In 58 cases (39.7% of patients with cirrhosis, respectively, 30.5% of all patients), alcoholic cirrhosis was described. 30 patients (20.5% of patients with cirrhosis, respectively, 15.8% of all patients) were described to have a non-alcoholic fatty liver disease, but the real number might be much higher. Six patients had hemochromatosis and three had autoimmune hepatitis. Some patients had multiple reasons for liver cirrhosis. 93 (48.9%) patients did not receive any therapy before iBT, and 97 (51.1%) already received another kind of therapy before. 35 (18.4%) received a TACE, 25 (13.2%) a resection, 24 (12.6%) were treated with sorafenib, 22 (11.6%) with a RFA, 20 (10.5%) received a selective internal radiation therapy (SIRT), 2 (1.1%) a portal-vein embolization, and 10 patients received other not specified therapies. 29 (15.3%) patients had a portal-vein thrombosis before the beginning of the therapy. 14 (7.4%) patients had one extrahepatic metastasis, and in 7 cases, metastases could not be ruled out. Most of the patients (115, 87.8%) were categorized as Child A and 16 (12.2%) as B. It was not possible to calculate the Child Score in 59 cases due to missing data. One patient was in the BCLC stage 0 (0.5%), 79 patients (41.8%) were in stage A, 74 (39.2%) in stage B, and 35 patients (18.5%) in stage C. It was not possible to determine the stage for one patient. 35 patients (18.4%) were treated with a two-timed iBT due to high tumor volume. 69 (36.3%) patients received an additional iBT due to progression, 14 (7.4%) received two iBT, and 18 (9.5%) received three or more. Median liver volume was 1482.5 cm^3^ and the clinical target volume was 48.5 cm^3^ (95% KI 31.37–75.72). Median age at iBT was 71.5 years (95% KI 68–74).

### Adverse events

In five cases, no information about complications during the therapy was documented. 10 (5.4%) patients had a minor-complication, three (1.6%) a major-complication, and one (0.5%) had a minor- and major-complication. 93 patients in total received an untimely follow-up, and in 64 cases, information about adverse events was recorded of which 16 patients had in total 19 adverse events. Therefore, 17% (considering 93 patients), respectively, 25% (considering 64 patients) had side effects. The most common adverse events were nausea, loss of appetite, and mild pain. The first scheduled follow-up after 3 months was accomplished in 160 patients and 118 had information about adverse events. 45 adverse events in a total number of 37 patients occurred leading to a rate of 23%, respectively, 31% of adverse events. Mild abdominal pain (16%), mild back pain (9%), and fatigue (7%) were documented most often. The data for follow-up-observation after 6, 10, and 13 months was similar. Altogether, 138 adverse events in 71 patients were reported in the follow-up observations until 13 months after iBT (634 observations at all and 420 with information about adverse events). The most common event was mild abdominal pain (16.7%) followed by nausea (10.1%), most with loss of appetite and fatigue (7.9%). Overall, there was no statistically significant correlation between pre-therapeutic ALBI or IBI scores and the occurrence of adverse events in follow-up observations or peritherapeutic complications.

### Overall survival

**Fig. 2:**
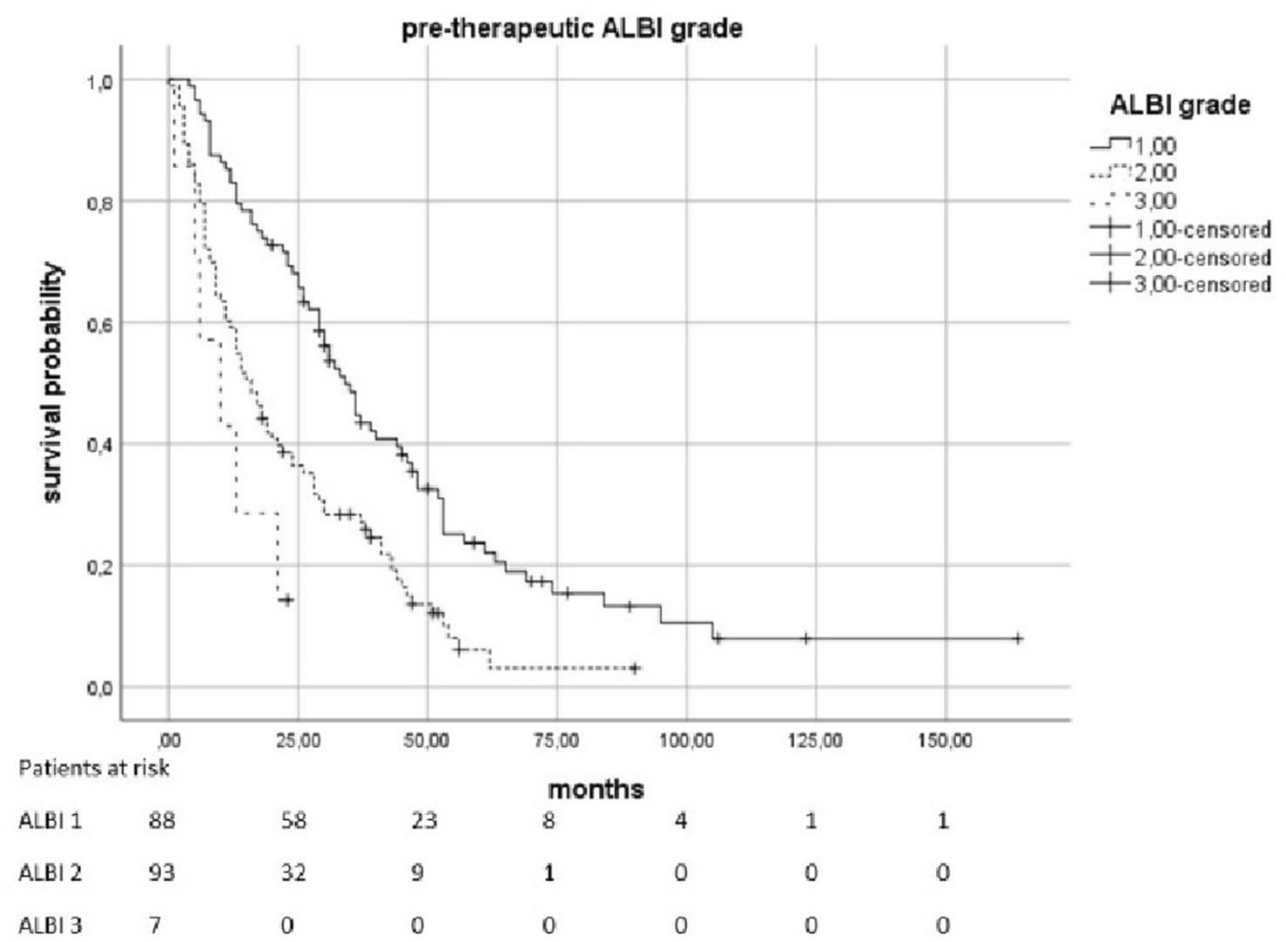
Overall survival according to the pre-therapeutic ALBI grade

**Fig. 3:**
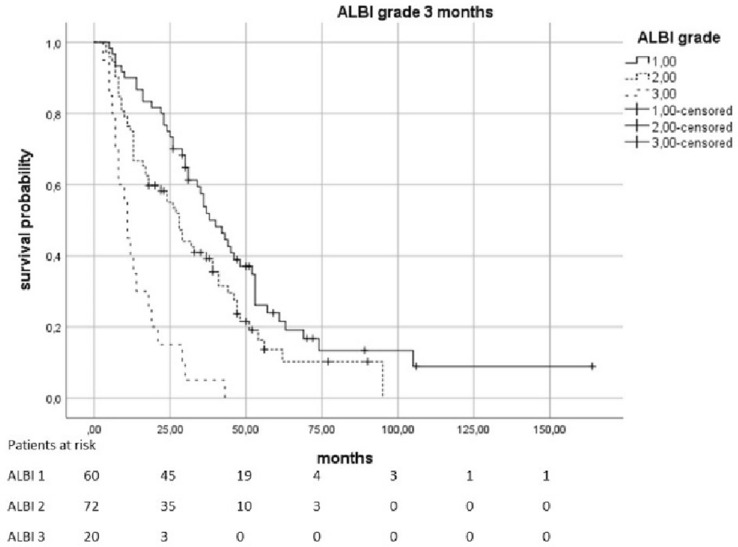
Overall survival according to the ALBI grade 3 months after therapy

**Fig. 4:**
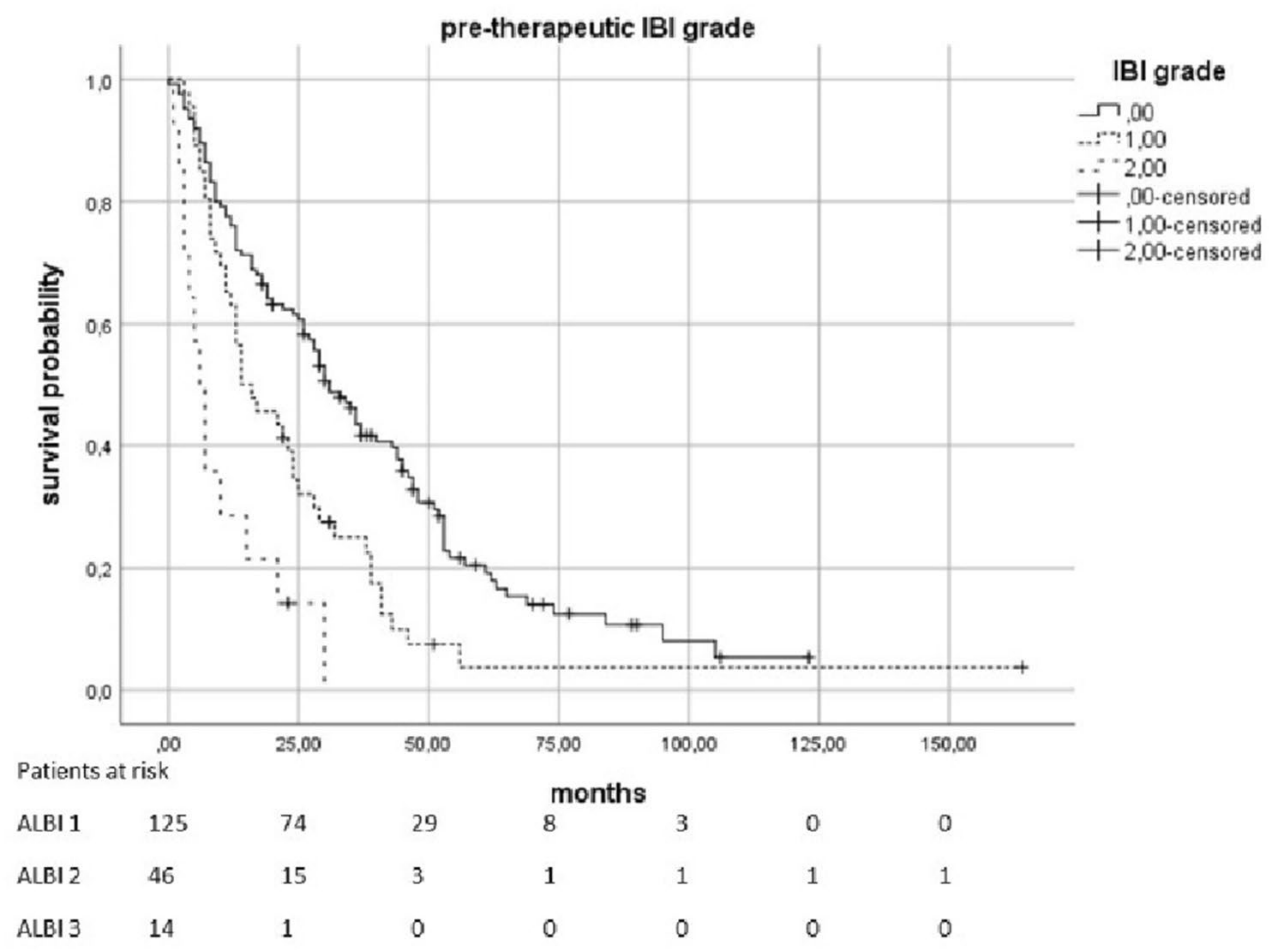
Overall survival according to the pre-therapeutic IBI grade

**Fig. 5:**
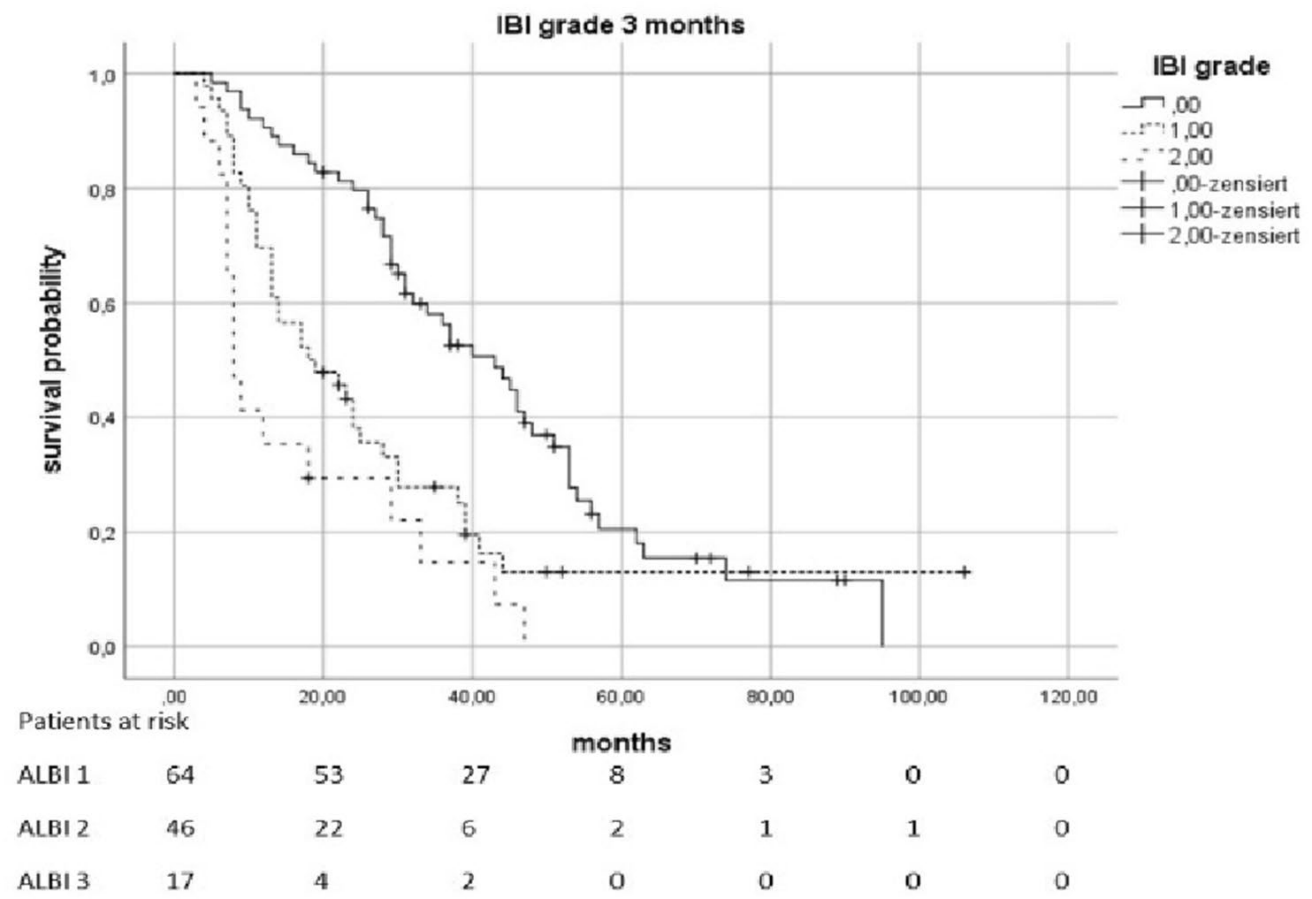
Overall survival according to the IBI grade 3 months after therapy

The median OS was 23.5 months (95% KI 19–28.5). Patients with a pre-therapeutic ALBI 2 had an increased risk of death by a factor of 2.01 compared to patients with ALBI 1 (*p* < 0.0021). In patients with a pre-therapeutic ALBI of 3, the risk was increased with a factor of 4082 (*p* = 0.0021). The median survival in patients with pre-therapeutic ALBI-Score 1 was 34 months (95% KI 28,398–39,602), in patients with a score of 2 was 16 months (95% KI 11,952–20,048), and in patients with ALBI 3 was only 10 months (95% KI 0–20,265). Patients with elevated pre-therapeutic IBI had a higher risk to die than the patients with IBI 0. IBI 1 vs. 0 increased the risk with the factor 1,816 (*p* = 0.0021) and 2 vs. 0 with the factor 4,608 (*p* < 0.0021). The median survival time for patients with IBI0 was 31 months (95% KI 25,179–36,821), 14 months (95% KI 5,138–22,862) for IBI 1, and 6 months (95% KI 3555–8445) for IBI 2 (Figs. 2, 3, 4 and 5).

Immediately after therapy only ALBI 3 vs. 1 showed a significantly higher risk with HR 4872 (*p* < 0.0021). The IBI-Score did also show significant differences between the groups after therapy with an HR of 3440 (*p* < 0.0021) for IBI 1 vs. 0 and an HR of 6210 (*p* < 0.0021) for IBI 2 vs. 0. The median survival of patients with a post-therapeutic IBI 0 was 62 months (95% KI 49,484–74,516), 24 months (95% KI 19,193–28,807) for IBI 1, and only 13 months (95% KI 10,917–15,083) for IBI 2.

Even 3 months after therapy, there were indications of a connection between ALBI-/IBI-Scores and OS. ALBI 2 vs. 1 causes an HR of 1556 (*p* = 0.0362) and 3 vs. 1 causes an HR of 5054 (*p* < 0.0021). IBI 1 vs. 0 was associated with an HR of 2018 (*p* = 0.0034) and 2 vs. 0 was associated with an HR of 3875 (*p* < 0.0021). The values of the following follow-up-examinations are shown in Table 2.

**Table 2:** Influence of ALBI and IBI on overall survival at different points in time

Time	Hazard’s ratio	*p* value	Median survival (95% KI) in months	Included cases
ALBI pre-therapeutic	2010 (2 vs. 1)	< 0.0021	1:34 (28,398–39,602)	188
	4082 (3 vs. 1)	0.0021	2:16 (11,952–20,048)	
			3:10 (0–20,265)	
IBI pre-therapeutic	1816 (1 vs. 0)	0.0021	0:31 (25,179–36,821)	185
	4608 (2 vs. 0)	< 0.0021	1:14 (5138–22,862)	
			2:6 (3555–8445)	
ALBI post-therapeutic	1655 (2 vs. 1)	0.1360	1: 36 (27,235–44,765)	166
			2: 26 (19,927–32,073)	
	4872 (3 vs. 1)	< 0.0021		
			3: 8 (4759–11,241)	
IBI post-therapeutic	3440 (1 vs. 0)	< 0.0021	0: 62 (49,484–74,516)	164
	6210 (2 vs. 0)	< 0.0021	1: 24 (19,193–28,807)	
			2: 13 (10,917–15,083)	
ALBI early FU	2160 (2 vs. 1)	0.0108	1: 31 (24,279–37,721)	88
	7257 (3 vs. 1)	< 0.0021	2: 18 (12,285–23,715)	
			3: 7 (5760–8,240)	
IBI early FU	2192 (1 vs. 0)	0.0256	0: 29 (24,422–33,578)	62
	2532 (2 vs. 0)	0.0133	1: 14 (9305–18,695)	
			2: 8 (6713–9,287)	
ALBI 3 months	1556 (2 vs. 1)	0.0362	1: 40 (31,987–48,013)	152
	5054 (3 vs. 1)	< 0.0021	2: 28 (22,635–33,365)	
			3: 11 (8820–13,180)	
IBI 3 months	2018 (1 vs. 0)	< 0.0034	0: 43 (32,894–53,106)	127
	3875 (2 vs. 0)	< 0.0021	1: 18 (8274–27,726)	
			2: 8 (1029–5983)	
ALBI 6 months	1876 (2 vs. 1)	0.0065	1: 40 (29,497–50,503)	135
	5863 (3 vs. 1)	< 0.0021	2: 28 (23,793–32,207)	
			3: 11 (7080–14,920)	
IBI 6 months	3144 (1 vs. 0)	< 0.0021	0: 46 (33,307–58,693)	101
	5273 (2 vs. 0)	< 0.0021	1: 17 (9857–24,143)	
			2: 11 (5456–16,544)	

We calculated differences in ALBI and IBI to evaluate dynamic changes as explained above. Pre- vs. post-therapeutic differences were only significant for IBI. A hazard ratio of 1792 (*p* = 0.0034) to have a worse OS was accompanied by an increasing IBI-Score. Increasing values between pre-therapeutic values and the 3 month follow-up increased the risk of death to 1870 (*p* = 0.0034) for ALBI and 1688 (*p* = 0.0256) for IBI. Decreasing values in ALBI showed a significantly higher OS with a hazard ratio of 0.649 (*p* = 0.0496) in comparison to patients with stable ALBI.

The Child Score showed a significant difference for patients with Child B vs. A with a hazard ratio of 2439 (*p* = 0.0021). No patients in our cohort were in the Child stage C. Only patients with BCLC-Stage C had a significantly lower OS compared to BCLC A. (HR: 2570 *p* < 0.0021). Between BCLC A and BCLC B patients, there was no significant difference in the OS.

### Progression-free survival

The median PFS was 7.5 months (95% KI 6–9). In comparison to the OS, the ALBI/IBI did not bring much additional information. After the Benjamini–Hochberg Correction, only ALBI 3 vs. 1.6 months after iBT and BCLC-Stage C vs. A showed a significantly higher risk for progress with hazard’s ratios of 2517 (*p* = 0.0465) and 2672 (*p* = 0.0310). Even without correcting the *p* values, the results were by far not as clear as the results for the OS.

## Discussion

Our results indicate that ALBI and IBI are valuable techniques for predicting the overall survival of patients with HCC treated with iBT. The pre-therapeutic ALBI-Score and follow-up examination scores demonstrated a strong connection with OS, and patients could be classified into several groups with markedly varied prognoses. Additionally, the IBI subdivided the cohort into several subgroups with widely disparate OS. The disparities across all groups were also statistically different in follow-up exams for ALBI and IBI. The information obtained by these scores may be beneficial in differentiating patients with a favorable or dismal prognosis, even if the commonly used Child–Pugh Score is incapable of doing so. Gkika et al. (2018) showed that patients with lower IBI had a higher OS when treated with stereotactic body irradiation, but not for ALBI or CP. One probable explanation for the variations in the research is that the patients had varying features. Our patients were mostly Child A patients with a smaller proportion of Child B patients, whereas Gkika et al. (2018) had a similar proportion of Child A and B patients. Additionally, our patients were evenly distributed across the BCLC subtypes, with 79 BCLC A, 75 BLCL B, and 35 BCLC C, whereas Gkika et al. patients were exclusively BCLC B and C. This indicates that the patients in Gkika et al. had more advanced tumors and worse liver functions than the patients in our analysis, which might be explained by the prior patient selection for iBT. This is also demonstrated by the median OS for patients with ALBI 1 at Gkika et al., which is only 17 months, compared to 34 months for our patients with pre-therapeutic ALBI 1. This implies that ALBI may be less effective in individuals with more advanced illness, although the lack of significance might also be explained by the smaller sample size. This hypothesis is further reinforced by the fact that Pinato et al. (2017) showed that the ALBI has an independent predictive value across all BCLC stages. ALBI and IBI were ineffective at predicting progression-free survival, as tumor progression may be more dependent on tumor stage than on liver function.

Our findings are limited by the study's retrospective nature and the large number of statistical tests, which results in a greater percentage of false-positive significant values. However, using the Benjamini–Hochberg approach, we were able to reduce the False Discovery Rate (FDR) to 5%, and the data clearly demonstrate a trend toward improved OS in patients with lower ALBI and IBI. We were not always able to demonstrate a significant difference between ALBI 2 and 3, or IBI 1 and 2, although this might be due to a small number of patients with ALBI 3 or IBI 2. Patient selection can potentially introduce bias, which is why the findings from this study cannot be generalized to the entire cohort of HCC patients. This study may serve as a starting point for prospective analysis to identify individuals who benefit significantly from iBT and those who may benefit more from other medicines or optimum supportive care. This should be investigated prospectively.

## Data Availability

The datasets used and analyzed during the current study are available from the first author on reasonable request.

## Abbreviations

HCC: Hepatocellular carcinoma
iBT: Interstitial brachytherapy
BCLC: Barcelona-clinic-liver-cancer
CP: Child Pugh
ALBI: Albumin–bilirubin grade
TACE: Transarterial chemoembolization
RFA: Radiofrequency ablation
OS: Overall survival
PFS: Progression-free survival
